# MIF Family Members Cooperatively Inhibit p53 Expression and Activity

**DOI:** 10.1371/journal.pone.0099795

**Published:** 2014-06-16

**Authors:** Stephanie E. Brock, Beatriz E. Rendon, Dan Xin, Kavitha Yaddanapudi, Robert A. Mitchell

**Affiliations:** Molecular Targets Program, James Graham Brown Cancer Center, University of Louisville, Louisville, Kentucky, United States of America; Weizmann Institute of Science, Israel

## Abstract

The tumor suppressor p53 is induced by genotoxic stress in both normal and transformed cells and serves to transcriptionally coordinate cell cycle checkpoint control and programmed cell death responses. Macrophage migration inhibitory factor (MIF) is an autocrine and paracrine acting cytokine/growth factor that promotes lung adenocarcinoma cell motility, anchorage-independence and neo-angiogenic potential. Several recent studies indicate that the only known homolog of MIF, D-dopachrome tautomerase (D-DT - also referred to as MIF-2), has functionally redundant activities with MIF and cooperatively promotes MIF-dependent pro-tumorigenic phenotypes. We now report that MIF and D-DT synergistically inhibit steady state p53 phosphorylation, stabilization and transcriptional activity in human lung adenocarcinoma cell lines. The combined loss of MIF and D-DT by siRNA leads to dramatically reduced cell cycle progression, anchorage independence, focus formation and increased programmed cell death when compared to individual loss of MIF or D-DT. Importantly, p53 mutant and p53 null lung adenocarcinoma cell lines were only nominally rescued from the cell growth effects of MIF/D-DT combined deficiency suggesting only a minor role for p53 in these transformed cell growth phenotypes. Finally, increased p53 activation was found to be independent of aberrantly activated AMP-activated protein kinase (AMPK) that occurs in response to MIF/D-DT-deficiency but is dependent on reactive oxygen species (ROS) that mediate aberrant AMPK activation in these cells. Combined, these findings suggest that both p53 wildtype and mutant human lung adenocarcinoma tumors rely on MIF family members for maximal cell growth and survival.

## Introduction

*p53* is the most commonly mutated tumor suppressor in human cancers, with a mutation rate higher than 50% [1]. Wildtype p53 protein is activated in response to cellular, genotoxic and oxidative stress and, following protein stabilization, serves to promote a transcriptional program that broadly attenuates malignant disease progression [1]–[3]. The p53 pathway is mutated at a high frequency in non-small cell lung carcinoma (NSCLC) lesions (50–70%), suggesting an important contribution to tumorigenic initiation and progression [4]. Those NSCLC lesions that harbor wildtype alleles of p53 are thought to have developed alternative mechanisms that serve to suppress p53 activity.

Macrophage migration inhibitory factor (MIF) is a pro-inflammatory cytokine that is overexpressed in a number of solid and hematologic malignancies with NSCLC being one of the highest overexpressing tumor types [5]. MIF promotes cell autonomous [6]–[9] and non-cell autonomous pro-tumorigenic processes [10]–[12]. However, several studies evaluating tumor initiation and maintenance in MIF-deficient settings reveal only modest decreases in tumor burden [13], [14]. Recent studies now indicate that the only other known MIF family member, D-DT functionally cooperates with, and compensates for, MIF in promoting neo-angiogenic potential in human NSCLC cells [6]. More recent studies demonstrate that MIF and D-DT additively antagonize the tumor suppressive activities of AMP-activated protein kinase (AMPK) in lung adenocarcinoma cells resulting in maximal mTOR pathway activation [15]. In this study, MIF and D-DT were found to additively promote glucose uptake/utilization resulting in enhanced glutathione reduction that, in turn, served to maintain low cellular oxidative stress. MIF and D-DT-deficient lung adenocarcinoma cells exhibit significantly less reduced glutathione levels and enhanced reactive oxygen species that were found to be necessary for the aberrantly activated AMPK observed in these cells.

MIF was first identified as a negative regulator of p53 by Hudson and colleagues using a functional p53 library screening assay [16]. Several studies have since validated MIF as being an important endogenous regulator of p53 expression and activity in a variety of biological processes [14], [17], [18]. A number of mechanistic pathways have been proposed for MIF-dependent p53 antagonism including: bioactive lipid metabolism [17], regulation of the COP9 signalosome subunit 5 (CSN5) [19], direct, physical interaction with p53 [20], indirect interaction with NM-23-H1 [21] and redox maintenance [22].

Because D-DT is an MIF compensating factor and is necessary for maximal MIF-dependent signaling in human lung adenocarcinoma cell lines [6], [15], we set out to determine whether D-DT functionally cooperates with MIF in modulating p53 expression and tumor suppressive activities in human lung adenocarcinoma cell lines. We now demonstrate that simultaneous, but not individual, siRNA knockdown of MIF and D-DT, results in a substantial induction of p53 phosphorylation, stabilization and activation of p53-dependent transcription in p53 wildtype NSCLC cell lines. MIF/D-DT-deficiency results in impaired cell growth phenotypes that were found to be only marginally dependent on aberrant p53 expression. Lastly, we demonstrate that aberrant p53 stabilization/activation observed in MIF/D-DT-deficient cells is independent of AMPK, a known p53 activator [23] and downstream effector of MIF/D-DT signaling [15], but is entirely dependent on enhanced reactive oxidative species (ROS). Collectively, our data indicate that MIF and D-DT cooperate to maintain low steady state p53 expression and activity in human NSCLC cell lines, and that this inhibition partially accounts for MIF/D-DT-dependent promotion of pro-growth phenotypes. Importantly, the functional overlap of MIF and D-DT in NSCLC pro-tumorigenic pathways provides strong rationale for the simultaneous therapeutic targeting of MIF and D-DT in lung adenocarcinoma malignant disease.

## Experimental Procedures

### Materials

N-Acetyl Cysteine (NAC), N-(2-mercaptopropionyl) glycine (MPG), crystal violet and propidium iodide solution were obtained from Sigma. 2′,7′-dichlorodihydro-fluorescein diacetate (H2DCFDA) was purchased from Invitrogen.

### Cell culture

NCI-H1299 (ATCC, Manassas, VA), A549 (ATCC) and A549-E6 (gift of Dr. Denise Galloway, Fred Hutchison Cancer Research Center) [29] cells were cultured in DMEM, 10% fetal calf serum, 2 mM glutamine, and 50 µg/mL Gentamicin. NCI-H838 and NCI-H460 (both from ATCC) cells were cultured in RPMI 1640 media supplemented with 10% fetal calf serum, 2 mM glutamine, and 50 µg/mL gentamicin.

### RNA Interference

Cells were transfected with MIF, D-DT, or nonspecific scrambled siRNA oligonucleotides using Oligofectamine reagent (Invitrogen) as previously described [6]. Commercially available siRNA oligonucleotides for human AMPK were purchased from Santa Cruz Biotechnology. Cells were incubated after siRNA transfection for the times indicated. Where indicated, shRNA knockdown of MIF and D-DT was achieved using specific shRNA lentiviral particles (Santa Cruz Biotechnology). Cells were infected at ∼50% confluency in the presence of Polybrene (Sigma). shRNA-expressing cells were selected for using puromycin. Control shRNA Lentiviral particles encoding a scrambled shRNA sequence were used as a negative control for these experiments.

### Immunoblotting studies

Whole cell extracts were prepared from cells after the indicated treatments. Cells were lysed in 1X lysis buffer (20 mM Tris, 137 mM NaCl, 1 mM EGTA, 1% Triton X-100, 10% glycerol, 1.5 mM MgCl2, 1 mM NaVO_4_, 2 mM NaF, and 1X protease inhibitor cocktail - Sigma) by repeated passages through a 27-gauge needle. Equal amounts of cellular protein were fractionated on SDS-polyacrylamide gels (Bio-Rad) and transferred to polyvinylidene difluoride (PVDF) membranes (Millipore). Immunoblotting was performed with antibodies directed against p53, MDM2, GAPDH, MIF, D-DT (Santa Cruz Biotechnology), AMPK, phospho-p53 (Ser15), phospho-Mdm2 (Cell Signaling Technology), p21 (BD Biosciences) and cleaved-PARP (Invitrogen). Densitometric analysis of western blots was performed using Bio-Rad Quantity One analysis software.

### Quantitative PCR Analysis

Total RNA was extracted from cells using the RNeasy Mini RNA extraction kit (Qiagen). cDNA was then synthesized from total RNA using the High Capacity Reverse Transcription Kit (Applied Biosystems) according to the manufacturer's instructions. Levels of MIF, D-DT and p21 mRNA were quantified from 5 ng of total cDNA using the TaqMan Individual Gene Expression Assay (Applied Biosystems). 18S rRNA expression was used as an internal control for analysis. Relative expression was determined using the ΔCt method.

### Adenovirus Preparation and Cell Infection

Adenovirus for human MIF and D-DT were prepared using the Gateway cloning system (Invitrogen). Briefly, human MIF and D-DT were PCR amplified and TOPO cloned into the pENTR/D-TOPO plasmid. MIF or D-DT inserts were shuttled into pAd/CMV/V5-DEST vector using LR recombinase, and subclones were confirmed by sequencing. Adenoviral vectors were digested with PacI, ethanol precipitated, and transfected into 293A adenoviral packaging cells using Lipofectamine. Virus was purified from viral supernatants using ViraBind purification columns (Cell Biolabs) and tested for expression efficiency versus toxicity. Cells were infected with virus at 70–80% confluence as indicated.

### Proliferation Assays

A direct cell count assay was performed after plating an equivalent number of shRNA lentiviral-infected cells into wells of a 12-well plate. Cells were plated in quadruplicate and enumerated by microscope every day for 4 days using 2 randomly chosen fields for each replicate. For ^3^H-Thymidine incorporation assays, 1×10^3^ siRNA-transfected cells were plated into each well of a 96-well plate and grown overnight. The following day, cells were pulsed with 1.5 µCi/mL ^3^H-Thymidine (MP Biomedicals) for 4 h, followed by vacuum transfer to a UniFilter-96 GF/C 96-well filter-bottomed plate (PerkinElmer). The radioactivity incorporated into the DNA was quantitated using a Packard TopCount-NXT microplate scintillation counter. Quantitation of ATP levels was also used to assess cell proliferation. Briefly, 1×10^3^ siRNA-transfected cells were plated into each well of a 96-well plate and grown for 48 h. The number of metabolically active cells was determined using the CellTiter-Glo Luminescent Cell Viability assay (Promega). ATP levels were determined by measuring luminescence according to the manufacturer's protocol.

For more stringent cellular proliferation analyses, soft agar and clonal proliferation assays were performed. Using 6 cm dishes for soft agar assays, 1×10^4^ siRNA-transfected cells in media containing 0.3% Noble agar (Difco) were layered on top of a solidified base of 0.6% Noble agar in media. Cells were fed every 3–4 days by adding 1 mL of 0.25% Noble agar in media. After 10–18 days, colonies were observed by staining with 0.005% crystal violet. For clonal proliferation assays, 500 siRNA-transfected cells were re-plated into each well of a 6-well plate. After 10–14 days of cell growth, foci were quantitated following staining with 0.005% crystal violet for 1 h. For inhibitor assays, 500 cells were plated into each well of 6 well plates and the following day, the indicated amount of 4-IPP or vehicle (DMSO) were added to each well.

### Apoptosis and Cell Cycle Analyses

For apoptotic analysis, the Annexin V:FITC Apoptosis Detection Kit (BD Biosciences) was used. Briefly, 1×10^5^ cells were incubated for 15 min in the presence of both 2.5 µL FITC Annexin and 10 µL propidium iodide in Binding buffer. Staining with FITC Annexin alone and propidium iodide alone, were used as controls. For cell cycle analysis, 1×10^6^ cells were fixed in 70% ethanol. The following day, the ethanol was removed from the fixed cells, followed by a 30-min incubation with propidium iodide (40 µg/mL). For both the apoptotic and cell cycle assays, the cells were immediately analyzed using a FACSCalibur flow cytometer (BD Biosciences). Data were analyzed and prepared using FlowJo software.

### Measurement of intracellular ROS

siRNA-transfected or 4-IPP-treated cells were lifted by trypsinization and washed with PBS. Cells were incubated with the ROS indicator H2DCFDA (5 µM) in PBS supplemented with CaCl_2_ and MgCl_2_. After a 30 min incubation at 37°C, cells were washed in PBS, and fluorescence was analyzed using a FACSCalibur flow cytometer (BD Biosciences). Data were analyzed and prepared using FlowJo software.

### Statistics

Results are expressed as means ±SD. Data comparisons were derived by one-way ANOVA when comparing groups of more than two and standard *t*-test when comparing groups of two using GraphPad Prism version 5.0. *p* values <0.05 were considered significant.

## Results

### MIF and D-DT cooperate to antagonize p53 expression and activation

**Figure 1:**
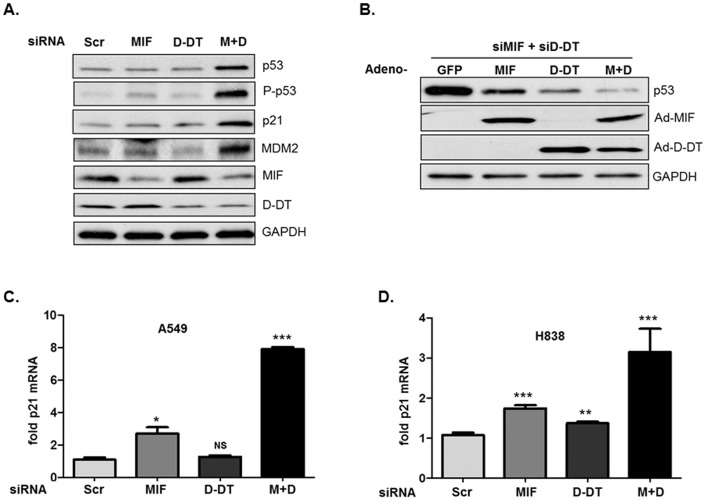
MIF and D-DT cooperatively inhibit p53 expression and activity. A: MIF and D-DT were silenced by siRNA transfection as indicated in A549 cells for 72 h followed by immunoblotting of cell lysates. Scr  =  scrambled oligos, M+D  =  MIF + D-DT siRNA oligos. B: A549 cells were transfected with siRNA as indicated for 48 h, followed by infection with GFP, MIF, D-DT, or MIF + D-DT (M+D) adenovirus overnight. Lysates were then analyzed by immunoblotting. A549 (C) or H838 (D) cells were siRNA-transfected as indicated for 72 h, and p21 transcript levels were determined using quantitative PCR. All data shown are representative of at least 3 experiments *, p<0.05; **, p<0.01; ***, p<0.001 by *t*-test analysis is shown for individual group comparisons to Scr control. NS  =  not significant.

Initial studies sought to determine whether D-DT recapitulates the documented suppressive activity of MIF against tumor suppressor p53. Using previously characterized and validated siRNA oligos targeting MIF and D-DT [6], [15], we knocked down both MIF family members - individually and jointly - and evaluated relative p53 responses in human lung adenocarcinoma cell lines. After validating efficient siRNA targeting of both MIF and D-DT at the protein (Fig. 1A) and mRNA levels (not shown), total and phospho-p53 (Ser15), and the p53 transcriptional targets p21 and MDM2 [24], [25], were evaluated by western blotting. As shown in Fig. 1A, individual siRNA knockdown of D-DT, similar to individual knockdown of MIF, had little effect on p53 phosphorylation (Ser-15), stabilization or transcriptional activation. However, simultaneous depletion of MIF and D-DT by siRNA resulted in a dramatic increase in the phosphorylation and stabilization of p53 concurrent with substantial increases in p53 target genes, p21 and Mdm2. Importantly, re-introduction of MIF and/or D-DT by adenoviral delivery effectively reversed the aberrant increase in p53 expression in MIF/D-DT-depleted cells (Fig 1B). This finding both validates the specificity, and rules out potential off-target effects, of MIF and D-DT targeting siRNA oligos.

p53 is a transcription factor that, when activated, results in enhanced transcription of its downstream targets [26]. The prototypical transcriptional target of p53 is the cyclin-dependent kinase inhibitor, p21 [24]. We next examined mRNA levels of p21 in MIF/D-DT-deficient conditions in order to validate the increased p21 protein levels observed (Fig. 1A). As shown in Figs. 1C and 1D, transcriptional expression of p21 was strongly induced following simultaneous knockdown of MIF and D-DT and only moderate p21 mRNA increases observed after individual MIF or D-DT knockdown in two independent p53 wildtype NSCLC cell lines. Collectively, these data indicate collaborative/compensatory functions for MIF and D-DT family members in NSCLC p53 modulation.

### MIF and D-DT deficiency results in defects in cell cycle progression and survival of NSCLC cells

**Figure 2:**
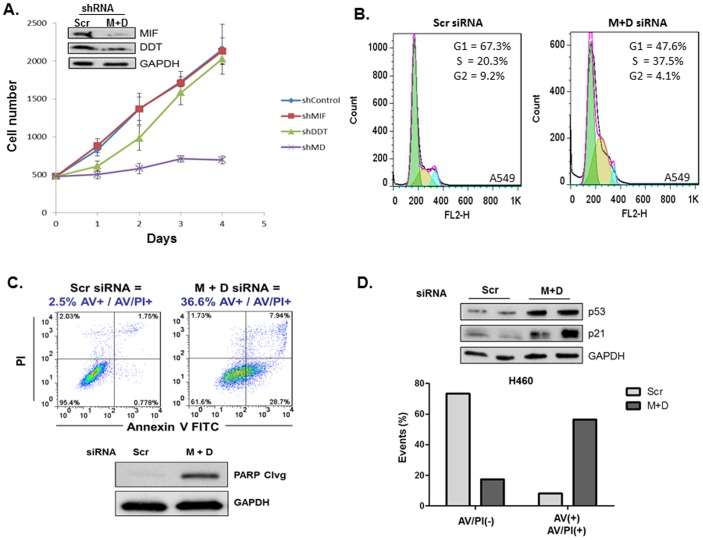
MIF and D-DT depletion results in defects in cellular proliferation, growth, and survival. A: A549 cells were infected with lentiviral Scr, MIF, D-DT or a combination of MIF + D-DT shRNA. After 72 h, an equivalent number of selected cells were plated in quadruplicate and enumerated for 4 days following plating. B,C: A549 cells were transfected with siRNA oligos as indicated for 96 h. Cell cycle distribution was assessed using FACS analysis of propidium iodide (PI) stained cells (B). Apoptosis was evaluated using FACS analysis of Annexin-V/PI-stained cells and immunodetection of cleaved PARP in lysates (C). D: H460 cells were transfected with siRNA oligos for 72 h followed by immunoblotting of lysates (top panel) or 96 h followed by FACs analysis of Annexin-V/PI-stained cells as in (B). Data shown are representative of 2 (A) or 3 (B,C) independent experiments. ***, p<0.001 by *t*-test analysis is indicated for individual group comparisons to Scr control.

The p53 protein is a master regulator of cell growth and survival responses that occur as a result of cellular stress [27]. Because of the observed MIF/D-DT-dependent regulation of p53 expression and activation, we next evaluated the phenotypic consequences of individual and combined MIF/D-DT-deficiency in lung adenocarcinoma cells. Lentiviral-delivered MIF and D-DT shRNA were introduced into A549 human lung adenocarcinoma cells, selected with puromycin, and then viable cells were plated and assessed for cell doubling by a simple cell counting assay. As shown in Fig. 2A, combined MIF/D-DT knockdown – but not individual knockdown – resulted in the nearly complete loss of cell growth and division. It is important to note that MIF/D-DT shRNA expressing cells were unable to be maintained and passaged after selection with puromycin and plating of the remaining viable cells in the cell counting assay. Because this cell growth phenotype was indicative of defective cell cycle progression, we evaluated cell cycle profiles of control and MIF/D-DT-deficient cells using propidium iodide DNA staining. As shown in Fig. 2B, MIF/D-DT-deficient cells exhibited an S-phase arrest profile which has previously been associated with p53 induction [28]. As MIF/D-DT shRNA expressing cells appeared to have significantly fewer viable cells compared to the nonsense control following infection/selection, we next evaluated whether there was any appreciable effect on cell survival following depletion of MIF and D-DT. As shown in Fig. 2D, significant increases in Annexin-V/PI staining (Fig. 2C – top panel) and a corresponding induction of poly (ADP-ribose) polymerase (PARP) cleavage (Fig. 2C – bottom panel) were observed in MIF/D-DT-deficient cells indicative of increased programmed cell death. Importantly, we observed similar effects of combined MIF/D-DT-deficiency on the induction of p53 and p21 (Fig. 2D – top panel), and on the apoptosis phenotype (Fig. 2D – bottom panel) in another p53 wildtype human lung adenocarcinoma cell line, H460. Combined, these data suggest that MIF and D-DT act in an additive and compensatory manner in promoting lung adenocarcinoma cell growth, division and survival.

### Phenotypic effects of MIF and D-DT deficiency are only partially dependent on the presence of p53

**Figure 3:**
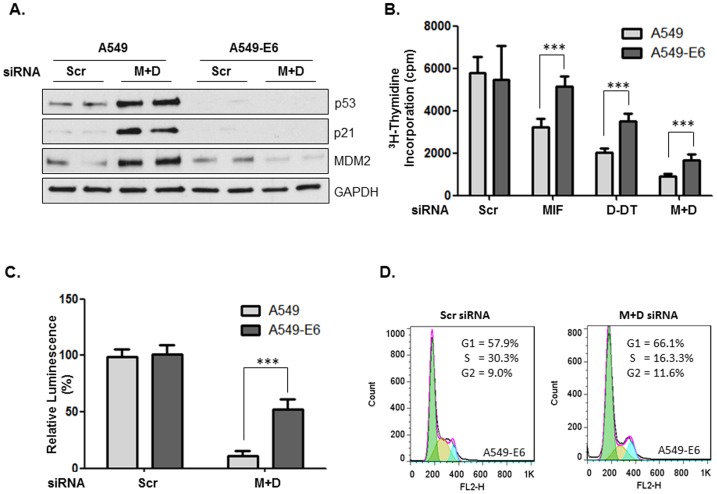
Phenotypic effects of MIF/D-DT depletion are only nominally dependent on p53. A: A549 and A549-E6 cells were transfected with siRNA oligos as indicated for 72h and lysates were analyzed by immunoblotting. B,C: MIF and/or D-DT were silenced by siRNA transfection as indicated in A549 or A549-E6 cells for 48 h, followed by re-plating into wells of a 96-well plate. Cell proliferation was assessed by a ^3^H-thymidine incorporation assay (B) and viability was assessed using the Cell-Titer Glo Assay (C). D: MIF and/or D-DT were silenced by siRNA transfection as indicated in A549-E6 cells for 96 h followed by FACS analysis of propidium iodide (PI) stained cells. Data shown are representative of 3 independent experiments. ***, p<0.001 by one-way ANOVA analysis is indicated for individual group comparisons.

In order to investigate whether the aberrant activation of the p53 pathway was responsible for the cell cycle progression defects observed in MIF/D-DT-deficient cells, we next utilized stably over-expressing human papillomaviral type 16 (HPV16) E6 oncoprotein A549 cells [29]. HPV16 and HPV18-encoded E6 protein forms stable complexes with cellular p53 resulting in p53 ubiquitylation and proteasomal degradation and rendering E6 oncoprotein-expressing cells p53-deficient [30]. E6-expressing A549 cells are well documented to be resistant to DNA damage-induced p53 activation and ensuing p21 expression [30]–[32]. As shown in Fig. 3A, A549-E6 cells exhibit no steady state or, more importantly, no MIF/D-DT-associated aberrantly expressed p53, p21 or MDM2, in stark contrast to parental A549 cells (Fig. 3A). Not only does this demonstrate that the p53 pathway is effectively disabled in E6-expressing A549 cells, it also supports the hypothesis that increased p21 and Mdm2 expression induced by loss of MIF and D-DT in A549 cells is, in fact, dependent upon functional p53 (Fig. 1). Interestingly, A549-E6 cells were only marginally – albeit significantly – able to reverse defective ^3^H-thymidine incorporation into DNA (Fig. 3B) and loss of cell viability (Fig. 3C) present in MIF, D-DT and MIF/D-DT-deficient cells. Importantly, the apparent S-phase arrest profile observed in MIF/D-DT-deficient A549 parental cells (Fig. 2B) was lost in A549-E6 cells (Fig. 3D), however, we did observe what appeared to be a moderate G1/S phase arrest in these cells. We tentatively hypothesize that this residual G1/S phase arrest observed in MIF/D-DT-deficient p53 null cells may account for the lack of a robust rescue of defective ^3^H-thymidine incorporation into DNA (Fig. 3B). Combined, these findings indicate that MIF/D-DT-dependent regulation of cell cycle and cell growth phenotypes is only nominally dependent on p53 antagonism in human lung adenocarcinoma cells.

### MIF and D-DT are functionally necessary for clonal proliferation

**Figure 4:**
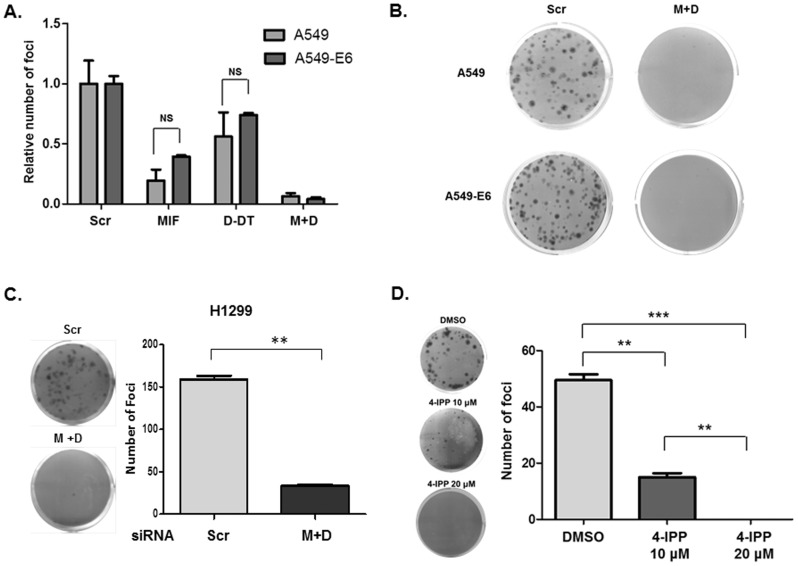
MIF and D-DT promote clonal cell proliferation independent of p53. A,B: A549 and A549-E6 cells were transfected with siRNA oligos as indicated for 48 h and then re-plated in duplicate in 6 well plates at 500 cells/well. 10–14 days later, colonies were stained with crystal violet, manually enumerated (A) and photographed (B). NS  =  not significant. (C) H1299 (p53 null) cells were transfected with siRNA oligos as indicated for 48 h and then re-plated in duplicate in 6 well plates at 1000 cells/well. 10–14 days later, colonies were stained with crystal violet, photographed (left panel) and manually quantified (right panel). D: A549 cells were plated in duplicate at 500 cells/well in 6 well plates in the presence of vehicle (0.1% DMSO), 10 µM 4-IPP or 20 µM 4-IPP. Vehicle and 4-IPP were replenished every other day. 10-14 days later, colonies were stained with crystal violet, photographed (left panel) and manually quantified (right panel). Data in all panels are representative of three independent experiments.

We next investigated the functional requirements for endogenous MIF and D-DT in a more stringent assay of clonal cell proliferation in A549 and A549-E6 cells. As shown in Figs. 4A and 4B, loss of MIF alone significantly reduced clonogenic growth while the combined loss of MIF and D-DT resulted in a nearly complete loss of colony formation. Also, consistent with the ineffectual rescue of ^3^H-thymidine incorporation and the residual G1/S phase arrest still present in MIF/D-DT-deficient A549-E6 cells (Figs. 3B and 3D, respectively), there was little to no difference in defective clonal proliferation between p53 wildtype and p53 null MIF/D-DT-deficient cells (Figs. 4A and 4B). To validate this finding and to ensure that p53 was, in fact, dispensable for MIF/D-DT-deficiency associated defects in colony formation, we utilized the p53 null human NSCLC cell line, H1299. Like E6-expressing A549 cells, H1299 cells are resistant to DNA damage-induced p53 stabilization and ensuing p53-dependent p21 transcription [31]. Similar to A549 and A549-E6 lines, H1299 cells exhibit significant defects in colony formation in response to simultaneous loss of MIF and D-DT and, to a much lesser extent, in response to individual MIF deficiency (Fig. 4C).

Prior studies from our laboratory identified and characterized a small molecule inhibitor of MIF, 4-iodo-6-phenylpyrimidine (4-IPP) that acts as a potent, irreversible, small molecule antagonist of MIF [33] and, to a lesser extent, D-DT (unpublished observations). We next sought to determine whether 4-IPP was able to recapitulate MIF and/or D-DT-deficiency in clonogenic growth assays. As shown in Figs. 4D, 4-IPP dose-dependently inhibited lung adenocarcinoma clonogenic focus formation in a manner that closely resembles MIF/D-DT-deficiency (Figs. 4A – 4C). These findings are reflective and in line with our prior studies demonstrating that 4-IPP inhibits anchorage-independent colony formation in human lung adenocarcinoma cell lines [33].

### AMPK is dispensable for aberrant p53 activation and clonal proliferative defects in MIF/D-DT-deficient cells

**Figure 5:**
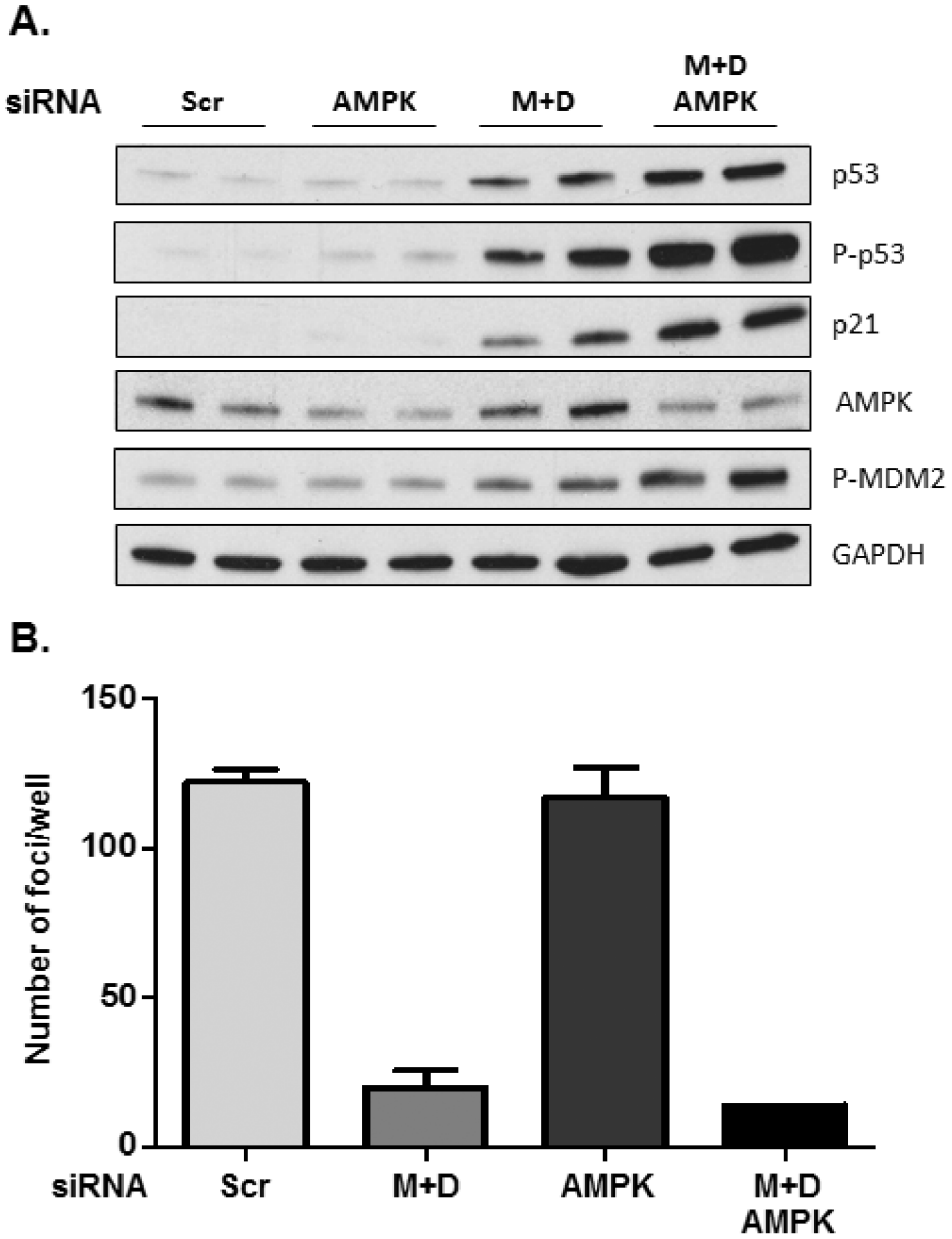
MIF/D-DT-dependent regulation of p53 and clonal proliferation are independent of AMPK. A: A549 cells were transfected with siRNA oligos as indicated for 72 h and cell lysates were analyzed by immunoblotting. B: A549 cells were transfected with siRNA oligos as indicated. After 48 h, 500 cells were plated in duplicate in 6 well plates. Colonies were stained with crystal violet and enumerated. Data shown are representative of two (B) or three (A) independent experiments.

We recently identified additive and redundant functions for NSCLC MIF and D-DT in maintaining low steady state AMPK activity [15]. Because AMPK induces tumor suppression, in part by facilitating p53 phosphorylation and stabilization [23], we next sought to determine whether aberrant AMPK activation in MIF/D-DT-deficient lung adenocarcinoma cells was responsible for the aberrant activation/stabilization of p53. Utilizing siRNA oligos against AMPKα (Fig. 5A) and a small molecule AMPK inhibitor, compound C (data not shown), our data indicate that reducing AMPK expression or inhibiting AMPK activity in MIF/D-DT-deficient cells has no inhibitory effect on p53 and, if anything, resulted in a slight enhancement of p53 phosphorylation and stabilization (Fig. 5A). We next assessed whether aberrantly activated AMPK in MIF/D-DT-deficient cells was involved in defective clonal cell proliferation of human NSCLC cell lines observed with MIF/D-DT-deficiency. As shown in Fig. 5B, no appreciable effect on clonal cell proliferation was observed with AMPK knockdown in MIF/D-DT-deficient cells suggesting that AMPK, like p53 (Figs. 4A and 4B), is dispensable for the defective proliferative phenotypes associated with loss of MIF family members. Combined, these results suggest that aberrantly activated AMPK in MIF/D-DT-deficient cells [15] is not solely responsible for the increased p53 activation or the defective clonal proliferation phenotypes observed in MIF/D-DT-deficient lung adenocarcinoma cells.

### Increased oxidative stress is responsible for p53 activation in MIF/D-DT-deficient cells

**Figure 6:**
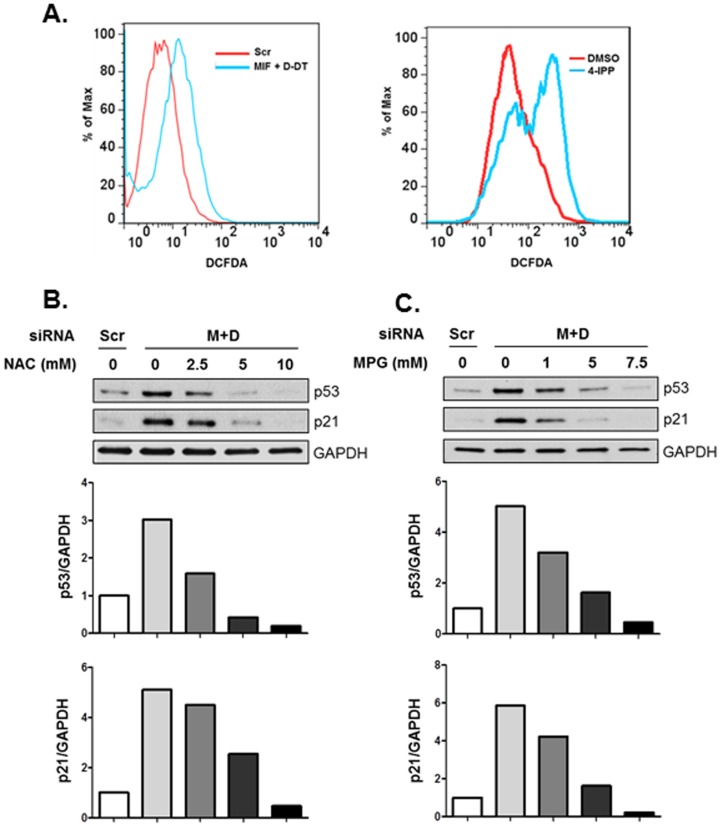
MIF and D-DT regulate p53 in a redox-dependent manner. A: A549 cells were transfected with siRNA oligos for 72 h or treated with 50 µM 4-IPP overnight as indicated. Intracellular ROS levels were assessed by flow cytometry upon incubation with the fluorescent ROS detector, DCF-DA. B,C: A549 cells were transfected with siRNA oligos as indicated. 48 h later, increasing concentrations of NAC (B) or MPG (C) were added to the cells for an additional 16 h. Lysates were analyzed by immunoblotting. Bio-Rad Quantity One software was used for densitometry and p53/GAPDH or p21/GAPDH densitometry values are depicted in the graph. Data shown are representative of four independent experiments.

Our prior studies investigating MIF family member contributions to nutrient metabolism in human NSCLC cells revealed that MIF and D-DT cooperatively promote glucose uptake and flux resulting in both cellular ATP homeostasis and maintenance of reduction-oxidation (redox) balance [15]. MIF and D-DT combined deficiency results in compromised cellular ATP/AMP ratios, lower levels of reduced glutathione and significantly increased levels of dichlorofluorescein (DCF)-detectable reactive oxygen species (ROS) [15]. Oxidative stress is a necessary consequence of cellular metabolism. When redox homeostasis is lost, proteins, lipids and DNA are all subject to oxidative damage [34]. Because p53 is activated by DNA damage initiated by oxidative stress [35], [36], we next sought to determine whether increased oxidative stress resulting from MIF/D-DT-deficiency might account for the aberrant p53 activation observed in these cells. After validating increased DCF-detectable oxidative stress in MIF/D-DT-deficient cells and 4-IPP treated cells (Figs. 6A), we tested two independent ROS scavenging compounds – N-acetylcysteine (NAC) and N-(2-mercaptopropionyl) glycine (MPG) – for relative inhibition of aberrantly expressed/activated p53 in MIF/D-DT-deficient cells. As shown in Figs. 6C and 6D, both NAC and MPG dose-dependently reversed aberrantly expressed p53 and p21 protein levels in MIF/D-DT-deficient cells suggesting an important role for MIF and D-DT family members in maintaining redox balance that, in turn, is necessary for preserving low steady state p53 pathway activation in p53 competent cells.

## Discussion

*p53* allelic mutation occurs in greater than 50% of primary NSCLC lesions and p53 mutational frequency correlates with morbidity in patients with malignant lung cancer [37]. While several studies have identified MIF as a negative regulator of p53 expression and activity, the data presented here are the first to describe a cooperative and compensating function for the MIF homolog, D-dopachrome tautomerase, in p53 maintenance. Importantly, functional cooperation by MIF and D-DT was found to extend to lung adenocarcinoma proliferative and clonal growth potential. While individual loss of MIF was observed to be generally dominant in generating defective clonal proliferation phenotypes, maximal defective growth and proliferation phenotypes were invariably observed when MIF and D-DT were simultaneously knocked down by siRNA. These findings suggest that simultaneous targeting of MIF and D-DT may have important, and previously unrecognized, therapeutic benefits for NSCLC patients. MIF, D-DT and their shared cell surface receptor, CD74, are over-expressed in human NSCLC [5], [6]. Both MIF and D-DT are functional ligands for cell surface-associated CD74 [38] and each can functionally compensate for the other to provide an autocrine signaling axis that serves to promote NSCLC angiogenic growth factor expression and maintain low steady state AMPK activation [6], [15]. Aberrant AMPK activation in MIF/D-DT-deficient NSCLC cells is due to a previously unrecognized additive role for MIF and D-DT ligands in maintaining redox homeostasis and ATP/AMP ratios via CD74-dependent-glucose uptake and subsequent metabolism [15]. Because AMPK promotes p53 phosphorylation [23] and aberrant p53 phosphorylation correlates, almost exactly, with aberrant AMPK activation in MIF/D-DT-deficient cells ([15] and *data not shown*), we initially speculated that AMPK was responsible for the observed increases in p53. However, we found no evidence of a role for AMPK in mediating aberrant p53 activation/stabilization in MIF/D-DT-deficient cells (Fig. 5A), nor did we find that aberrant AMPK contributed significantly to MIF/D-DT-associated proliferative defects (Fig. 5B). We now provide evidence, though, that the increased levels of ROS – likely H_2_O_2_ [15] – present in MIF/D-DT-deficient cells [15] (Fig. 6A) are responsible for increases in p53 phosphorylation, stabilization and ensuing p53-dependent transcription in MIF/D-DT-deficient cells (Fig. 6). Our prior studies suggested that defective glucose uptake as a consequence of MIF and D-DT loss results in reduced pentose phosphate shunt activity that, in turn, leads to less NADPH-dependent glutathione reduction and increased hydrogen peroxide [15]. Although beyond the scope of this current study, it is highly likely that p53 activation in MIF/D-DT-deficient NSCLC cells is secondary to a DNA damage response initiated by oxidative stress present in these cells (Fig. 6A).

Our studies indicate that combined loss of MIF and D-DT results in cell survival (Fig. 2) and cell proliferative defects (Figs. 3, 4) that are largely p53-independent (Figs. 3, 4). Additionally, we found no evidence that AMPK was a significant contributor to MIF/D-DT-deficiency associated defective clonal cell proliferation in p53 wildtype cells (Fig. 5B) or, from preliminary studies (*not shown*), in p53 null cells either. Although studies are currently underway to determine the more general involvement of prolonged oxidative stress to defective clonal cell proliferation in these cells, the more practical explanation may be that the culmination of defects in metabolic, oxidative and proliferative signaling pathways present in MIF/D-DT-deficient NSCLC cells [15], [33], [39] is simply not compatible with malignant cell growth. Lastly, during the preparation of this manuscript, a study by Pasupuleti and colleagues was published identifying a similar additive and compensatory requirement for MIF and D-DT in controlling cell growth and survival properties of human renal cell carcinoma cell lines [40]. Interestingly, p27 cyclin-dependent kinase inhibitor was very recently identified as being aberrantly up-regulated in response to MIF/D-DT knockdown adding yet another important cell growth/survival pathway of regulation by MIF family members [40].

Highly unique among cytokines and growth factors, MIF and D-DT share an evolutionarily conserved vestigial catalytic activity involved in the tautomerization of D-dopachrome, a product of tyrosine catabolism not naturally found in mammals [41]–[43]. Importantly, MIF also has a tautomerase-independent thiol-protein oxidoreductase catalytic activity requiring cysteines 57 and 60 within the MIF polypeptide [44]. Because D-DT lacks a cysteine at position 60 (serine), it is highly unlikely that MIF-associated protein oxidoreductase activity is involved in the oxidative stress observed in MIF/D-DT-deficient cells. Mutation or small molecule targeting of MIF's catalytic active site blocks MIFs bioactivities – perhaps by impeding residues within the substrate binding pocket that have been conserved to participate in binding to MIF and D-DTs' shared cell surface receptor, CD74 [33], [45]. Our initial attempts to recapitulate MIF/D-DT-deficiency using small molecule MIF antagonists indicate that the irreversible MIF inhibitor, 4-iodo-6-phenylpyrimidine (4-IPP) [33], strongly inhibits NSCLC clonal proliferation in a manner similar to that observed in MIF/D-DT-deficient cells. Moreover, 4-IPP induces significant increases in ROS (Fig. 6A) and decreases in glucose uptake ([15] and not shown) in a manner that essentially recapitulates MIF/D-DT-deficiency. When taken together, these studies provide compelling rationale for developing dual targeting MIF/D-DT small molecule inhibitors as novel NSCLC therapeutic agents.

In summary, our studies indicate that MIF and D-DT provide additive and redundant functions in maintaining p53 tumor suppressor levels in human lung adenocarcinoma cells. We further demonstrate that MIF and D-DT cooperatively promote NSCLC proliferative potential in a largely p53-independent manner. Future MIF targeting strategies should take into consideration the fact that, for maximal anti-NSCLC therapeutic efficacy, both MIF family members should be targeted simultaneously.
